# Social determinants of pulmonary tuberculosis treatment non-adherence in Rio de Janeiro, Brazil

**DOI:** 10.1371/journal.pone.0190578

**Published:** 2018-01-05

**Authors:** Elvira Maria Godinho de Seixas Maciel, Juliana de Souza Amancio, Daniel Barros de Castro, José Ueleres Braga

**Affiliations:** 1 Sérgio Arouca National School of Public Health (Escola Nacional de Saúde Pública Sérgio Arouca), FIOCRUZ, Rio de Janeiro, Brazil; 2 Health Surveillance Foundation (Fundação de Vigilância em Saúde, FVS), Manaus, Brazil; 3 Institute of Social Medicine (Instituto de Medicina Social), Rio de Janeiro State University (Universidade do Estado do Rio de Janeiro, UERJ), Rio de Janeiro, Brazil; University of Liverpool, Institute of Infection & Global Health, UNITED KINGDOM

## Abstract

Success in tuberculosis control depends on the implementation of steps that reduce social inequities, allowing the diagnosis and effective treatment of the disease. Little is known about the conditions affecting antituberculosis treatment non-adherence in areas of great social and economic heterogeneity, such as the municipality of Rio de Janeiro. This study aimed to describe and identify the social determinants of antituberculosis treatment non-adherence in the municipality of Rio de Janeiro between 2008 and 2012. An ecological study was conducted with the districts of Rio de Janeiro as the units of analysis. Analyzes using Poisson regression models allowed us to identify the association between dropout from antituberculosis treatment and the human development index and social development index. The final model showed that economic conditions, infrastructure, and the tuberculosis control quality of surveillance were associated with treatment non-adherence. This study demonstrated that the scenarios of socio-environmental precariousness found in the districts of Rio de Janeiro were able to identify populations with an increased risk of default treatment from antituberculosis.

## Introduction

Rio de Janeiro is one of the five Brazilian state capitals with the highest incidence of tuberculosis in recent years [1–3]. Control of this endemic disease requires reducing social inequalities and improving access and coverage to tuberculosis-related health services [4].

According to the World Health Organization, Brazil is among 22 countries that are responsible for 80% of all the cases of tuberculosis, ranking 16th in 2014 [5]. Each year there are approximately 70,000 new cases of tuberculosis, corresponding to an incidence rate of 35 cases per 100,000 inhabitants. Despite a reduction over the last 15 years, the number of cases of tuberculosis in Brazil is currently almost static. In recent years, the state of Rio de Janeiro has almost doubled the country's incidence (60 cases per 100,000 inhabitants) and also experienced a slowdown in the reduction of the morbidity burden. The municipality of Rio de Janeiro has higher incidence rates (80 cases per 100,000) than the state average but in this city, there has been a reversal of the incidence trend with an increase over recent years unlike the state and the country. Non-adherence to treatment represents an important barrier to treatment effectiveness and it affects 11%, 12% and 14% of patients with TB undergoing treatment in the country, state and municipality, respectively, in the last decade [1–3]. Therefore, strategies to control tuberculosis incidence in the state of Rio de Janeiro should prioritize the municipality of Rio de Janeiro.

Considering the transmission of the disease, the existence of effective treatment, and the absence of a protective vaccine for the pulmonary form, the National Tuberculosis Control Program (NTCP) proposes, as the main control step, the timely identification of cases and the effective treatment of the disease [6]. It is known that the effectiveness of the treatment depends on a combination of the use of adequate drugs, and correct doses for sufficient time. Since the 1970s, a standardized six-month scheme has been used in Brazil. Treatment non-adherence persists as a major challenge to the effectiveness of tuberculosis treatment. As a key strategy for treatment success, the NTCP recommends directly observed treatment (DOT), an element of the directed Observed Therapy Strategy (DOTS) aiming to strengthen patient adherence to treatment. Considering the other components of the strategy, it is important to emphasize that in addition to directly observed treatment, measures should be taken to reduce social vulnerability.

In the last decade, after the implementation of DOTS, the municipality of Rio de Janeiro presented little variation in the proportion of treatment non-adherence. The treatment non-adherence rate was approximately 14%, in 2005 and 13% in 2014 [2]. This scenario indicates the need for establishing the causes of treatment non-adherence. Studies suggest that in addition to individual characteristics, precarious social conditions may influence compliance with tuberculosis treatment [7–9]. There is considerable research on individual factors associated with treatment withdrawal and on the social determinants of tuberculosis risk, while there are few studies examining the social determinants of treatment non-adherence.

Nonadherence with tuberculosis treatment adversely affects its control, maintaining the level of transmission of the disease, and favoring the occurrence of multidrug-resistant tuberculosis. It appears that reducing the incidence relies on the adoption of more specific and effective knowledge-based measures affecting the determinants of noncompliance with treatment [4]. Rio de Janeiro has significant social and economic heterogeneity and considerable differences in the proportion of nonadherence with tuberculosis treatment, and it is reasonable to suppose that such differences may be associated with the distribution of its social determinants. The objective of this study was to describe and identify the social determinants of non-adherence to tuberculosis treatment in the municipality of Rio de Janeiro between 2008 and 2012.

## Materials and methods

**Fig 1:**
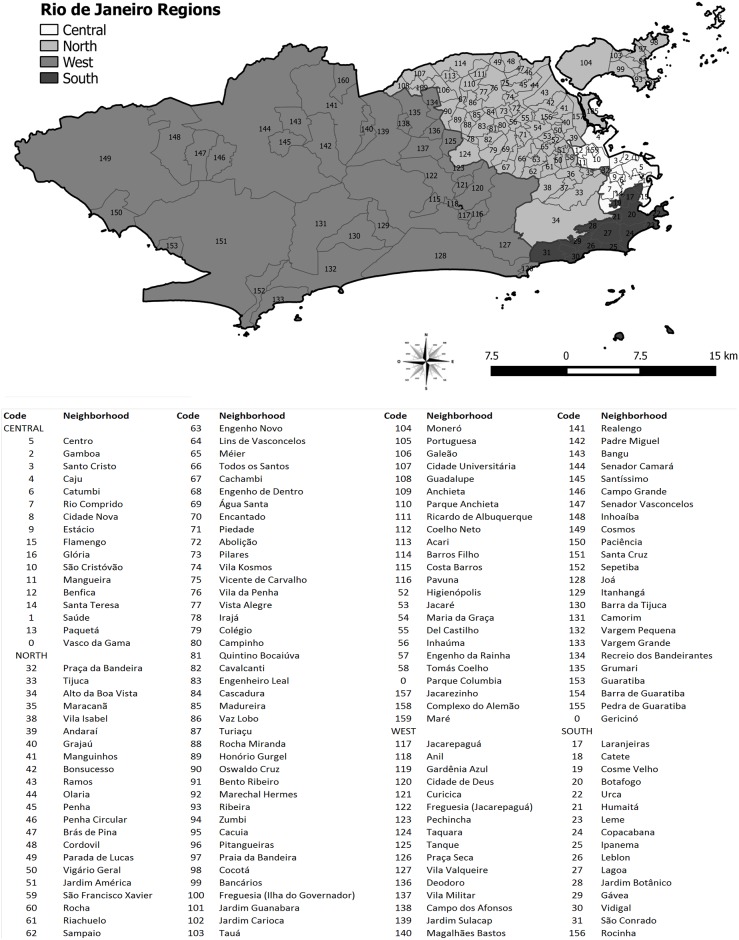
Regions and neighborhoods of the municipality of Rio de Janeiro.

An ecological study was conducted, based on the neighborhoods of the municipality of Rio de Janeiro as analysis units. Located at latitude 22°54′10″ S and longitude 43°12′27″ W, in 2010, the municipality of Rio de Janeiro, capital of the state of Rio de Janeiro, was reported to cover an area of 1,197 km^2^ composed of 160 neighborhoods and a population of 6,320,446 inhabitants [10]. In the present study, Vasco da Gama, Gericinó, and Parque Colúmbia neighborhoods, newly created from the division of the territories of São Cristóvão, Bangu, and Pavuna, respectively, were kept in the neighborhoods of origin (Fig 1).

We included all new cases of pulmonary tuberculosis notified between 2008 and 2012 and affecting residents of the municipality of Rio de Janeiro. New cases of tuberculosis were defined as follows, according to the recommendations of the Brazilian Ministry of Health: all patients with sputum smear microscopy positive for *Mycobacterium tuberculosis* or a clinical history of tuberculosis associated with results of complementary tests, such as radiological findings, and who never underwent anti-tuberculosis treatment or were treated for up to 30 days. Non-adherence to treatment was defined as the patient’s inability or refusal to take tuberculosis medication as prescribed by the health professional [11,12]. Duplicate records and those with no information on the patient's address were excluded [6].

Data from patients with tuberculosis were obtained from the Notification of Injury Information System. The data on the resident population and the socioeconomic information of the districts of Rio de Janeiro were obtained from the demographic census conducted in 2010 by the Brazilian Institute of Geography and Statistics and the Social Development Index (SDI), used to represent the socioeconomic situation of the studied districts. This data was obtained from the Municipal Institute of Urbanization Pereira Passos (IUPP) of the city hall of Rio de Janeiro.

The SDI is a composite index calculated from ten indicators obtained from the 2010 demographic census and considers four dimensions: (i) access to basic sanitation; (ii) housing quality; (iii) degree of schooling; and (iv) availability of income. In order to represent the access dimension to basic sanitation, the following indicators were used: (a) proportion of households with adequate water supply services, defined as those with internal pipelines and connected to the general network; (b) proportion of households with adequate sewage service, defined as those that are connected to the general network; and (c) proportion of households with adequate garbage collection services, defined as those with direct or indirect collection of garbage. The income availability dimension was calculated by (a) the average income of the household heads on minimum salaries; (b) the proportion of household heads with income up to two minimum wages, and (c) the proportion of heads of household with income equal to or greater than 10 minimum wages. The average number of toilets per person was used as a proxy for the dimension of housing quality, and the educational level was determined by the following: (a) the proportion of illiteracy among persons over 15 years; (b) proportion of household heads with less than four years of schooling; and (c) the proportion of household heads with 15 years or more of schooling. To obtain the SDI, the IUPP team standardized each of the indicators using the formula shown below:

$$I^{S}=\frac{I-(min(I)-0,01)}{max\left(I\right)-(min(I)-0,01)}$$

Where I is the value of an indicator for a neighborhood, max (I) is the maximum value of indicator I among all neighborhoods, min (I) is the lowest value of indicator I among all neighborhoods, and I^S^ is the standardized value of indicator I. The arithmetic mean of the standardized indicators was then calculated.

In addition to the SDI, other composite indicators traditionally used in studies of social determinants were analyzed: (a) the human development index (HDI); (b) the human development index education dimension (HDI-e); (c) the human development index longevity dimension (HDI-1); and (d) the human development index income dimension (HDI-i).

Simple indicators were analyzed representing the following dimensions: (i) social, (ii) economic, (iii) infrastructure, and (iv) quality of tuberculosis surveillance and control. The following indicators were used as a proxy of the social condition: (a) life expectancy at birth, (b) the illiteracy rate of the population aged 18 years and over, and (c) the proportion of the population living in households with more than two residents per dormitory. The economic condition was represented by: (a) ratio between the average income of the richest 20% and the poorest 40%; (b) the income Gini index; (c) the proportion of the extremely poor (*per capita* household income equal to or less than R$ 70.00 monthly); (d) proportion of the poor (*per capita* household income equal to or less than R$ 140.00 monthly) and (e) *per capita* income (ratio between the sum of the income of all individuals living in permanent private households and the total number of individuals). The following indicators were used as a proxy for neighborhood infrastructure: (a) the proportion of the population living in households with bathrooms and water, (b) the proportion of the population living in households with a garbage collection service, and (c) the proportion of the population living in households with electric energy. The indicators representing the performance of tuberculosis control services were calculated from the available information contained in the records of patients with tuberculosis, as follows: (a) the proportion of new cases of pulmonary tuberculosis with laboratory confirmation, (b) the proportion of new cases of cured pulmonary tuberculosis (closed by cure), (c) the proportion of new cases of smear-positive pulmonary tuberculosis in directly observed treatment, and (d) the proportion of new cases of pulmonary tuberculosis reported in the neighborhood of residence. These are indicators of monitoring of tuberculosis surveillance and control services recommended by the NTCP [13].

An exploratory analysis performed using boxplots and thematic maps, in order to verify the possible associations between the social determinants and the non-adherence to treatment of pulmonary tuberculosis. In this step, the composite indicators HDI and SDI were categorized. The HDI was classified as very low (0–0.499), low (0.500–0.599), medium (0.600–0.699), high (0.700–0.799), and very high (0.800–1). SDI values were grouped by quartiles and classified as low, medium, high, and very high.

Poisson regression models were used to identify the social determinants of non-adherence with antituberculosis treatment. Firstly, a bivariate analysis was performed to examine the association between each of the indicators studied and the nonadherence with anti-tuberculosis treatment. Then, multivariable analysis was performed with the variables of each dimension. Using the stepwise backward method, we selected the variables that showed an association with the outcome at a level of significance less than or equal to 0.2. To obtain the final model, the variables selected in the previous step were analyzed in a multivariable Poisson regression model. Variables showing an association with outcome at a level of significance less than or equal to 0.05 were selected.

Statistical analyzes were performed using the STATA version 13 application (StataCorp, College Station, Texas, USA) and the thematic maps were constructed using the QGIS software version 2.12.3 (OSGeo, Beaverton, OR, USA). Regarding the ethical aspects of this research, the study used secondary data of unrestricted access, made available under the authorization of government agencies, and obtained approval from the Committee of Ethics in Research of the National School of Public Health Sergio Arouca—Oswaldo Cruz Foundation on December 08, 2015, registry CAAE 49428515.3.0000.5240.

## Results

Between 2008 to 2012, the proportion of cases of noncompliance with treatment was 13.8% in the municipality of Rio de Janeiro. In some districts, such as Vila Militar and Campo dos Afonsos, there were no cases of treatment nonadherence. Conversely, in Manguinhos and Barra de Guaratiba districts the proportion of cases with treatment non-adherence was 26% and 29%, respectively.

**Fig 2:**
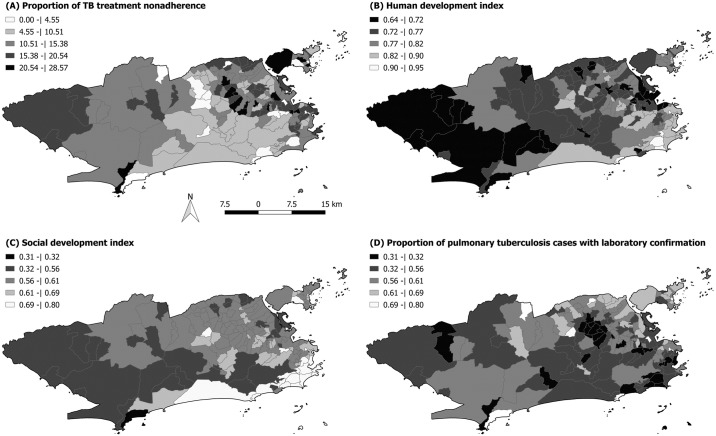
Spatial distribution of (A) proportion of anti-tuberculosis treatment non-adherence, (B) HDI, (C) SDI, in the districts of the municipality of Rio de Janeiro.

Fig 2A shows the spatial distribution of the dropout ratio of tuberculosis treatment. Most districts had a high level of treatment non-adherence (above 5%). There was a higher concentration of districts with a high proportion of treatment non-adherence in the North, Center, and West regions of the municipality, while in the Southern region treatment non-adherence rates were lower. Coincidentally, all the neighborhoods in the South region had a very high HDI, except for Rocinha (0.662) and Vidigal (0.756), as shown in Fig 2B. The Benfica neighborhood, located in the Central region, presented a lower HDI index (0.621) than mean HDI. The neighborhoods in the Southern region also presented the highest SDI values (Fig 2C), with the exception of the neighborhoods Rocinha (0.54) and Vidigal (0.57). The highest SDI value (0.8 in the Lagoa neighborhood) was observed in this region. The lowest SDI values were found in Grumari (0.31) and Vargem Grande (0.45), both located in the West Region of Rio de Janeiro.

**Fig 3:**
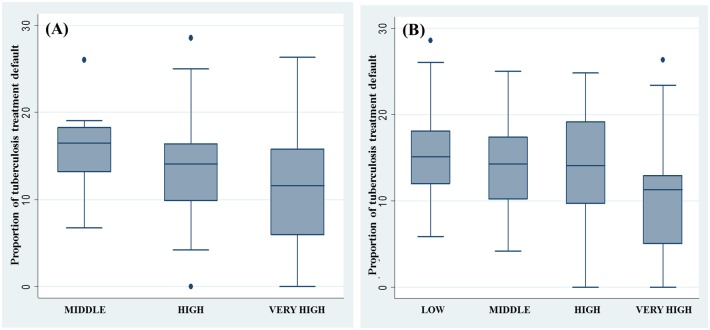
Boxplot of the proportion of non-adherence to antituberculosis treatment by strata of socioeconomic status: (A) human development index and (b) social development index.

The distribution of the dropout rate for tuberculosis according to HDI levels shows that, on average, dropout rates are lower in districts with high and very high HDI (Fig 3A). In the districts with high HDI, extreme treatment non-adherence values were observed: Guaratiba bar (HDI 0.761; treatment non-adherence 28.6%), Paquetá and Vila Militar (HDI 0.789 and 0.777, respectively; each with 0% dropout rates). The stratification of the neighborhoods according to the SDI showed that in the neighborhoods with the highest levels of social development, the proportion of noncompliance with antituberculosis treatment was lower, although i there is a considerable variability in treatment non-adherence in this group of districts (Fig 3B).

The composite indicators of development (SDI and HDI) were inversely associated with the proportion of cases of noncompliance with treatment non-adherence for pulmonary tuberculosis (Table 1) with a stronger association observed with the SDI than with the HDI. The HDI indicators specific to income, education, and longevity dimensions were also inversely associated with non-adherence with treatment and the association with the longevity dimension was stronger.

**Table 1:** Rate ratio of nonadherence with antituberculosis treatment for human and social development indicators according to the Poisson regression model.

Level of development	Rate Ratio	P value	IC 95%
**Composite indicator**			
Social Development Index—SDI	0,080	0,000	0,037–0,173
Human Development Index—HDI	0,156	0,000	0,080–0,306
Human Development Index (*education*)—HDI-e	0,293	0,000	0,170–0,502
Human Development Index (*longevity*)—HDI-l	0,041	0,000	0,013–0,123
Human Development Index (*income*)—HDI-r	0,166	0,000	0,092–0,302

Determinants of each analyzed dimension (social, economic, infrastructure, and quality of tuberculosis surveillance) were independently associated with noncompliance (Table 2). In the social dimension group, bivariate analysis revealed a direct association between treatment non-adherence and illiteracy and demographic density. Life expectancy at birth was inversely related to treatment non-adherence. In the multivariable regression model, the only variable significantly associated with treatment non-adherence was life expectancy at birth (p = 0.05). Regarding the economic dimension, there was a direct association between the proportion of the poor and the proportion of the extremely poor and treatment non-adherence. The other explanatory variables were inversely related to treatment non-adherence and the strongest association was observed with the income Gini index. Multivariate analysis revealed that only two variables remained in the final model: income *per capita* and the proportion of extremely poor. In the category of factors related to housing infrastructure, all variables were inversely associated with the treatment non-adherence, and in multivariable regression analysis, significant associations (p <0.05) were observed for all variables. The proportion of households with electricity was more strongly associated with treatment non-adherence, with an adjusted rate ratio of 0.270. Among the factors indicative of the quality of tuberculosis surveillance and control in the districts of Rio de Janeiro, the proportion of reported cases in the neighborhood of residence was directly associated with outcome, the cure rate was inversely associated with treatment non adherence, but it was not possible to detect an association between the others factors and treatment non-adherence.

**Table 2:** Rates of antituberculosis treatment non-adherence by social, economic, infrastructure, and health service quality factors, according to the Poisson regression model.

Factors	Crude rate ratio	IC 95%	Adjusted rate ratio	IC 95%
**Social conditions**				
Illiteracy rate—population ≥18 years old	1,040	1,015–1,065		
Life expectancy at birth	0,948	0,931–0,966	0,948	0,931–0,967
Demographic density	1,011	1,007–1,016		
**Economic conditions**				
*Per capita* income	0,999	0,999–0,999	0,999	0,999–0,999
Proportion of poor	1,036	1,023–1,050		
Proportion of extremely poor	1,105	1,060–1,152	1,066	1,015–1,120
*Per capita* Gini income	0,048	0,010–0,230		
20% richer / 40% poorer	0,914	0,873–0,958		
**Infrastructure**				
Proportion of households with bathroom and water in the residence	0,961	0,935–0,987	0,969	0,940–0,999
Proportion of households with garbage collection	0,929	0,898–0,960	0,945	0,910–0,981
Proportion of households with electricity	0,308	0,155–0,611	0,270	0,133–0,548
**Quality surveillance**				
Proportion of smear-positive lung cases treated DOTS	0,999	0,997–1.001		
Proportion of reported cases in the neighborhood of residence	1,002	1,001–1,003		
Proportion of cases confirmed by laboratory	0,997	0,994–1,001		
Proportion of cured cases	0,980	0,976–0,984	0,981	0,977–0,985

Poisson multivariable regression analysis using the variables selected from each dimension allowed us to verify the association between treatment non-adherence and factors related to the economy, infrastructure, and quality of tuberculosis surveillance (Table 3). The level of treatment non-adherence is directly associated with the proportion of extremely poor population, even adjusted for the proportion of households with electricity and the proportion of cases cured, with a protective effect for the outcome. The adjusted R^2^ of the final model was 0.26.

**Table 3:** Rate ratio of the selected variables of each dimension for the tuberculosis treatment non-adherence according to the Poisson multivariable regression model.

Factors/Indicators	Rate Ratio—Adjusted for Dimension	IC 95%	Rate Ratio—Adjusted Final Model	IC 95%
**Social conditions**				
Life expectancy at birth	0,948	0,931–0,966		
**Economic conditions**				
*Per capita* income	0,999	0,999–0,999		
Proportion of extremely poor	1,066	1,015–1,120	1,077	1,030–1,125
**Infrastructure**				
Proportion of households with electricity	0,969	0,940–0,999		
Proportion of households with garbage collection	0,945	0,910–0,981		
Proportion of households with electricity	0,270	0,133–0,548	0,346	0,170–0,700
**Quality surveillance**				
Proportion of cured cases	0,981	0,977–0,985	0,979	0,974–0,983

## Discussion

This study demonstrated the association between aspects of social and economic development and anti-tuberculosis treatment non-adherence in the municipality of Rio de Janeiro. In addition to the identification of causal associations, this approach sought to identify population living conditions capable of predicting failures in the health services that should perform actions to minimize the social inequalities on health.

Research has traditionally focused on studying the factors associated with the cessation of antituberculosis treatment, including patient characteristics and those relating to the functioning of services from the perspective of individual risks [14]. This approach, encouraged by international organizations, allowed the recognition of "more vulnerable" population groups, without engaging with the complexity of the scenarios that "predispose" to the treatment default in diagnosed cases [5]. The identification of individuals at higher risk of antituberculosis treatment non-adherence, such as indigenous people, street people, prisoners, and illicit drug users, indicates that strategies should be directed towards these specific groups. However, the knowledge developed in this area seems not to have been sufficient to reduce the level of treatment non-adherence in heterogeneous and complex urban contexts, such as that in large Brazilian metropolises, specifically Rio de Janeiro.

Our study found that human development was inversely related to the proportion of treatment non-adherence in the districts of Rio de Janeiro. The HDI, which includes income, longevity, and education, considers population characteristics associated with access to goods and services in general, which, in this context, could translate into a greater supply of better quality health services. It is important to note the strong association detected between the HDI of education and treatment non-adherence. In the economic context, education is generally analyzed in terms of its direct association with labor productivity, contributing to the growth of the economy [15]. However, it is also important to evaluate education in the social context, in terms of its potential to expand work opportunities, facilitate social mobility, and reduce inequalities [16].

It was also observed that the SDI was more strongly associated with treatment non-adherence than the HDI. This is probably due to the fact that the four dimensions that make up the SDI include ten indicators, ranging from access to basic sanitation to income, to quality of housing and schooling. Furthermore, the SDI evaluates living conditions in urban spaces, including those related to urban growth and its repercussions on the infrastructure and supply of basic services, which presupposes the need to use such indicators in the planning and management of the Municipality [17]. With regard to the treatment of pulmonary tuberculosis, the responsibility lies with the basic health care network. It is therefore an indicator that considers both the conditions related to human development and the urban characteristics that determine the quality of life of the residents, which would have a greater impact on treatment non-adherence.

As for the social dimension, both the illiteracy rate and the demographic density were related to the treatment non-adherence. Several studies have corroborated our findings, indicating that the level of schooling may affect the level of knowledge and the ability to seek compatible living conditions with a good level of health [18–21]. Urban agglomeration has been identified as a predictor of both the occurrence of tuberculosis and the undesired outcomes of its treatment, including neglect [22,23]. Life expectancy at birth was inversely associated with the occurrence of treatment non-adherence. This measure is considered to be an indicator of more comprehensive health conditions and was the only variable to remain independently associated in the joint analysis with the other indicators of social dimension. This reflects how life expectancy at birth is able to express the social conditions of a population.

Regarding the economic dimension, two indicators were directly associated with treatment non-adherence: the proportion of the poor and the proportion of the extremely poor. These findings are plausible, as the frequency of the poor seems to indicate difficulties in accessing good quality services, which can be taken as an indirect sign of the absence or inefficiency of State action. This poverty condition is known to be associated with other unfavorable conditions, such as schooling, access to healthy food, information, and employment [24]. *Per capita* income was inversely associated with treatment non-adherence. Numerous studies have identified the association between income, living conditions, and access to health services [25–27]. In our scenario, *per capita* income may represent access to services that are better qualified to treat tuberculosis. Two indicators of relative poverty, the Gini of *per capita* income and the ratio of rich to poor were also inversely associated with treatment non-adherence. The first indicator was more strongly associated with treatment non-adherence, reflecting the importance of economic inequality in the access to goods and services in general, and, specifically, in relation to health. The ratio between the rich and the poor also indicates, in the same direction, the association between economic inequality and treatment non-adherence. In multivariate analysis, only two indicators of economic conditions (*per capita* income and extremely poor proportion) remained independently associated with treatment non-adherence. Income distribution inequality (Gini index and ratio between rich and poor) and the proportion of poor indicators lose statistical significance in the multivariable model. This indicates that the effect of these conditions on treatment non-adherence can be explained by income conditions that remain in final model. This is likely to occur because these indicators express similar aspects of reality due to the similarity of the constructs.

Regarding the infrastructure dimension, the three indicators were inversely associated with treatment non-adherence. The existence of adequate sanitary conditions indicated a lower level of non-adherence to antituberculosis treatment. These indicators strongly reflect the presence of the State in the districts of the municipality of Rio de Janeiro. It makes sense that populations lacking adequate infrastructure are also affected by other conditions resulting in deficiencies in the treatment of tuberculosis. It should be emphasized that it is not the presence of a bathroom or water in the home that is the indicator of the causal association of the occurrence of treatment non-adherence, but this would be a predictive factor of conditions of care for individuals with tuberculosis. In the multivariate context, the occurrence of electricity in households remained an independent predictor of treatment non-adherence, probably because this indicator represents more strongly the indigence conditions of the population, consistent with the great level of iniquity [28].

Regarding the quality dimension of tuberculosis surveillance, there was no association between non-adherence to tuberculosis treatment and the proportion of cases with supervised treatment or laboratory confirmation tests. Conversely, a direct association was observed between the proportion of cases reported in the neighborhood of residence and treatment non-adherence. These results indicate that inefficiency in the organization of health services constitutes a greater obstacle to access to tuberculosis control strategies than the distribution of health units in the municipality of Rio de Janeiro. It is possible that this result is associated with low coverage of the Family Health Program (PSF), which is the main program responsible for supervised treatment, with a coverage of 3.5% in 2009, achieving 48% coverage in 2015 [29]. A further possible explanation could be violence in the coverage areas of some health units. For example, in a study conducted in a health planning area in the municipality of Rio de Janeiro, Paula and Aguiar [30], cited violence as an obstacle to the continuity of treatment. The authors describe that family health teams encountered difficulties performing their routine activities, such as active patient search. Furthermore, there were difficulties for users to move within the neighborhood, impairing access and use of health services offered; similar considerations apply to supervised treatment. The proportion of treatment non-adherence was inversely correlated with the proportion of cases of cured pulmonary tuberculosis, and this was significant in the multivariable analysis. This indicator reflects the deepening of the relationship between health professionals and users of the health network, revealing when strategies for tuberculosis control are effective for diagnosis, follow-up, and treatment. Conversely, the lack of ties between health professionals and users encourages treatment non-adherence.

The final model of this study showed that economic conditions, infrastructure, and surveillance were related to the treatment non-adherence. The following are the indicators that best predict treatment non-adherence represent more precarious conditions: the proportion of extremely poor, the proportion of households with electricity and the proportion of cured cases. We can affirm that this set of indicators, by corroborating the relationship between development—social and human—and treatment non-adherence, also offers a more specific alternative to classic composite indicators capable of identifying situations with a tendency to abandon them.

Although this study was the first study to use a comprehensive model of social determinants of treatment non-adherence in the municipality of Rio de Janeiro, the choice of neighborhoods as a unit of analysis may represent a limitation, as for some of the indicators studied, a level of internal heterogeneity to the unit that can influence the results. The rationale for the choice of this unit of analysis was that this is the smallest administrative unit for which public actions are planned at the municipal level. Regarding the quality of the data, a critical evaluation of the follow-up data of the treated cases was made, and the temporal cut allowed a high proportion of treatment results recorded in the database (SINAN-TB). Another important aspect is that a small proportion, less than 2% of the cases during the study period, could not be georeferenced.

In addition to the ability to identify scenarios with a higher propensity for treatment non-adherence, these findings highlight that conditions of social and economic development also affect the conditions of tuberculosis treatment among the population of Rio de Janeiro. Kehr (2016) [31] observed that tuberculosis control, including ensuring full treatment of marginalized populations, depends not only on the availability of drugs and the functioning of health services, but also on social and political inclusion. Mason (2016) [32] reaffirmed the importance of social science and medical anthropology concepts in the understanding of tuberculosis, stressing the need to recognize the complexity of the social, economic, cultural, geographic, and political aspects involved in the control of tuberculosis[33], In an ethnographic study of the abandonment of tuberculosis treatment among Bolivians Aymara, Greene (2004) recognized that in addition to cultural differences, social determinants of access to treatment can reduce tuberculosis control.

The knowledge produced here can support the elaboration of strategies aimed at strengthening and improving the basic health network, the expansion and qualification of basic care, and the expansion of supervised treatment, so that populations more vulnerable to treatment non-adherence can be assisted by in a targeted way. The identification of unfavorable conditions makes us think that other health outcomes may also be influenced by socio-environmental precariousness, and it draws attention to the role of the State, particularly with regard to the implementation of public policies with the objective of reducing the inequities in access to adequate living conditions and favorable to health.

## Conclusions

In conclusion, the antituberculosis treatment non-adherence in the municipality of Rio de Janeiro is determined by social development, human development, and social, economic, and environmental conditions capable of identifying populations more prone to the risk of treatment non-adherence.
